# Do people with different sociodemographic backgrounds value their health differently? Evaluating the role of positional objectivity

**DOI:** 10.3389/fpubh.2023.1234320

**Published:** 2023-12-13

**Authors:** Gaurav Jyani, Shankar Prinja, Aarti Goyal, Basant Garg, Manmeet Kaur, Sandeep Grover

**Affiliations:** ^1^Department of Community Medicine and School of Public Health, Postgraduate Institute of Medical Education and Research (PGIMER), Chandigarh, India; ^2^National Health Authority, Ministry of Health and Family Welfare, Government of India, New Delhi, India; ^3^Department of Psychiatry, Postgraduate Institute of Medical Education and Research (PGIMER), Chandigarh, India

**Keywords:** valuation of health, health inequity, health state preference, sociodemographic factors, socio-economic status, equity, positional objectivity, time-trade off

## Abstract

**Objective:**

The fundamental disconnect between the actual and the perceived health of an individual raises considerable skepticism on the self-reported health data as it may be confounded by an individual’s socio-economic status. In this light, the present study aims to assess if people with different sociodemographic backgrounds value their health differently.

**Methods:**

The health-state valuation using time-trade off was performed in a cross-sectional survey among a representative sample of 2,311 adults from India. Individuals were selected using a multistage stratified random sampling from five Indian states to elicit their present health-state, and to perform the health-state valuation exercise using computer assisted personal interviewing. A single block of standardized health-states was valued by multiple individuals, each belonging to different socio-demographic group. The difference in the valuation of health was assessed using bivariate analysis. The impact of different sociodemographic factors on the health-state valuation was evaluated using Tobit regression model.

**Results:**

Differences in the valuation of health were observed among different groups of age, religion, family type, state of residence, substance abuse, presence of ailments at the time of valuation, and number of dependent members in the household. Even after controlling for the severity of the administered health states, factors having a significant association with the valuation of health are age, religion, state of residence, substance abuse, family type, number of dependent members in the household, and presence of chronic or both acute and chronic ailments. Younger individuals place a higher value to their health as compared to their older counterparts. As compared to a healthy individual, a person with ailments rates the same health-state as worse.

**Conclusion:**

Inequalities in self-reported ill-health cannot be attributed to positional objectivity; age, religion, state of residence, substance abuse, family type, dependents, and ailments impact individual health valuation.

## Introduction

One of the best indicators of the well-being of a society is its health status (1–3). When considered at a broad level, the health status conveys the information about the general wellness, productivity, and development of a society (4). A comparison of health status across different sub-groups of a population offers valuable insights into their differential health needs and to understand its determinants in order to undertake corrective measures (5, 6). The information on health status can be obtained from various sources like self-reported health-status surveys, disease burden studies, scrutiny of hospital records, death registers, etc. Among these sources, self-reported health/morbidity is one of the commonest methods used in countrywide surveys to assess the health status of a population. This information is then used extensively in assessment of health needs of different population groups while formulating health policies and programs (7, 8).

Although self-reported health has a good face-validity, problems arise when comparisons are made across different sub-groups of the population (9). Notably, self-reported health surveys in India indicate that wealthier individuals tend to report more illnesses than their less affluent counterparts (10, 11). This contradicts objective measures such as life expectancy or mortality rates, which typically show higher affliction rates among the less privileged (7, 8, 10). It raises considerable skepticism on the self-reported measures of health and morbidities.

One potential reason behind this dichotomy can be the concept of positional objectivity, which postulates that what we observe depends on our position with respect to the objects of observation (12). In other words, it articulates that despite being in the same health-state, an individual living in better socio-economic context places a different value to his/her health as compared to the poorer counterpart. Likewise, some sections of the society may be more aware and conscious about their health status and thus in a better position to appraise their health than others. This may also be linked to their ability to seek care for even minor ailments due to their ability to pay. It has been reported that there is a fundamental disconnect between the actual (objective/clinically determined) health state of an individual and the perception (subjective/self-reported) of one’s own health (13, 14). This is because an individual’s assessment of own health is heavily contingent upon its socio-demographic context and consequent social experiences (12).

An alternative interpretation of the seemingly paradoxical observation of greater self-reported ill-health among the affluent might be influenced by the stage of epidemiological transition that a society is undergoing (10). During the initial phases of transition, as non-communicable diseases are on the rise, affluent individuals are more susceptible than their less affluent counterparts, primarily due to lifestyle practices (15). This suggests that the observed disparity in self-reported morbidities, with elevated rates among the rich, could be reasonable or plausible. In contrast to these studies, the recent evidence suggests that the poor report more illness as compared to the rich (16). There is also a regional variation in the extent of this unequal and disproportionately higher morbidity among the poor. In a way, this evidence challenges the significance of positional objectivity, or at least it suggests that the impact of positional objectivity is insufficient to counterbalance the biological influence of social determinants, resulting in increased morbidity among the less privileged.

In this context, it is imperative to conduct a thorough investigation into the disjunction between an individual’s subjective evaluation of their health and the objectively determined health status. This exploration is crucial due to its far-reaching implications for the fundamental concept of health equity and its subsequent influence on patterns of healthcare utilization. An in-depth examination is required to unravel the complexities surrounding individuals’ perceived health and its alignment with objective health status. For example, the hospitalization services in India are being utilized at a higher rate by the high socio-economic groups (17, 18). If it is correct that the richer population truly undergoes greater rates of morbidity than the poorer population, the elevated utilization of health services by the wealthy may not be inherently inequitable. However, if the hypothesis of positional objectivity holds true, implying that those with higher socio-economic status tend to exaggerate their health issues, while individuals with lower socio-economic status tend to downplay or underreport their health problems, the observed inequity in utilization might be even more pronounced than what is being currently observed (19). Hence, an urgent need arises to investigate whether individuals with different sociodemographic backgrounds attribute distinct values to their health.

One of the ways to assess this question empirically is to present standardized descriptions of cases or clinical vignettes or health-states to individuals with different socio-economic status and observe their valuation of health. Considering the widespread use of self-reported health data in designing public policies, it becomes crucial to tackle this issue (7, 8). Nevertheless, the majority of efforts to evaluate the current state of health in India have relied on the self-reported health information that people have provided, failing to take into account the possibility that people in varying socioeconomic strata may have differential preferences in experiencing and reporting their health (20).

Furthermore, it has been observed in the realm of economic evaluations that their outcomes are very sensitive to the utility values of the health states which are considered in such assessments (21, 22). This is because the utility values assigned to different health states act as a critical parameter in these evaluations, significantly influencing the overall results and conclusions drawn. The sensitivity of these outcomes emphasizes the importance of accurately determining and understanding the utility values associated with diverse health conditions (23–25). It underscores the need for precision and reliability in the valuation process, as any variations in the assigned utility values can significantly impact the economic evaluation outcomes, potentially altering decisions related to resource allocation, policy formulation, and healthcare planning. Given that these utility values are also determined through health valuation among patient population, there arises a compelling need to investigate whether individuals hailing from diverse socio-demographic backgrounds attribute distinct value to their health and convey these values dissimilarly. Nonetheless, there has been no previous evaluation of this impact by presenting individuals from diverse socioeconomic backgrounds with a uniform health condition and soliciting their assessments. Consequently, this study carried out an empirical assessment among the Indian population to determine whether individuals from various socioeconomic backgrounds place different values on their health and report it differently. Therefore, we used the health-state valuation data obtained from a representative sample from India to assess how sociodemographic background of an individual influences the value which a person confers to his/her health.

## Materials and methods

### Study settings and sampling

To obtain a sample representative of the country’s population, the sample selection involved a rigorous process wherein the selection was made at five different levels, i.e., at the level of states, districts, primary sampling units (PSUs), households, and the individuals to be interviewed. The health-state valuation data were collected in five states of India, selected based on three criteria: income, health status, and geographical representation. The states thus selected were—Haryana, Uttar Pradesh, Gujarat, Odisha, and Tamil Nadu. Two districts were selected from each of these states, considering their relative performance on the indicators of education, health, and living standards (26). In order to select the sample within a district, “30-cluster sampling approach” recommended by the World Health Organization (WHO) was used, taking care of the rural-urban distribution of the Indian population (27). Within a cluster (primary sampling unit), the respondents were selected using a multistage stratified random sampling technique. The detailed sampling approach has been published separately (28).

To have valid regional level estimates, sample size was first estimated at the state level. As the data were collected using EQ-5D-5L instrument, the health-related quality of life scores obtained from EQ-5D-5L (utility scores) for all health states were considered as the main variable of interest. Using the standard deviation of this variable (SD = 0.53), and assuming absolute precision (*d*) as 0.05 and a 95% confidence interval, a sample size of 435 was estimated (29). As the study was conducted in five different states, a minimum sample of 2,175 comprising all five states was considered appropriate for the study. The health state valuation data for assessing the impact of socio-demographic factors on valuation of health-states were collected alongside the development of an EQ-5D Value set for India using an Extended design (DEVINE) study (28, 30).

### Study instruments and valuation methods

The EQ-5D-5L instrument was used to record the present health-state of the respondent and to perform the health-state valuation exercise. The EQ-5D is the most commonly used instrument worldwide to measure health-related quality of life (31). Developed by the EuroQol Group in the 1980s, it serves as a succinct and generic instrument designed for measuring, comparing, and valuing health status across various disease areas (32). It is a standardized measure of health-related quality of life that assesses an individual’s health across five dimensions: mobility, self-care, usual activities, pain/discomfort, and anxiety/depression. The “5 L” in EQ-5D-5L signifies that each dimension has five possible levels of response, i.e., no problems, slight problems, moderate problems, severe problems, and extreme problems (33). Based on the level of difficulty reported by the individual among the five dimensions, an EQ-5D-5L health state is defined for it, which is represented as a five-digit number (e.g., 11111, 11112, etc.), wherein each digit represents the level of problem in the respective dimension. The computer assisted personal interviewing (CAPI) technique using EuroQol Group’s Valuation Technology (EQ-VT) software of the latest available version 2.1 was used to interview the participants (34–36). After recording the present health-state of the respondent, health state valuation exercise was performed with the respondent using time-trade off (TTO) experiment.

For the health-state valuation, the respondent was presented a predetermined hypothetical EQ-5D-5L health-state and was asked to imagine himself/herself in this health-state. The respondent was then asked to compare and value this health-state by juxtaposing it with an alternative health-state of perfect health using TTO. In TTO valuation, the respondent was asked to indicate the amount of time he/she was willing to give up to attain perfect health. The respondents were asked if they prefer to live for 10 years in perfect health (life A) or 10 years in the given (inferior) health state (life B). The time available in life B was kept constant at 10 years, while the time available in life A was changed sequentially as per the preference of the respondent, and the respondent was asked to select the better alternative between life A and life B. In other words, the respondent was asked to state its preference between “living for 10 years in an inferior health state,” and “living for less than 10 years in perfect health.” This exercise was performed till the point of indifference is achieved (when the respondent felt that both life A and life B were of equal value). At this point of indifference, the traded-off time in life A was recorded, which reflected the time in perfect health the respondent was willing to give up to avoid living in the inferior health state (life B). Using this iteration, the value of the given health-state was calculated as *x*/*t*, where “*x*” is the time remaining in life A at the point of indifference, and “*t*” is the time offered in life B, i.e., 10 years (35).

Each respondent performed the valuation of one block of health-states, which contained 10 predetermined health-states. The study utilized 18 such blocks of 10 health-states, comprising 150 unique health states in total (37). Each block included one most severe health state (55,555) as anchor state, and one of the five very mild health states (which demonstrates slight problem in any one of the five dimensions, i.e., 11112, 11121, 11211, 12111, and 21111). The remaining eight unique health states in each block (in total 144 health states in 18 blocks) were selected using Monte Carlo simulations (28, 34).

In addition to recording the self-reported health status of the respondent and performing 10 health-state valuation exercises, the data on socio-demographic characteristics of the respondent were also collected using a structured questionnaire. As the health-state valuation exercise is very sensitive to the interviewer’s effect, comprehensive trainings were organized, followed by an extensive phase of pilot interviews. Additionally, stringent quality control measures were practiced throughout the study (28, 38). The recommendations of the latest EQ-VT protocol were followed to standardize the data collection process across different regions of the country (34, 39, 40). The EuroQol Group provided English as well as officially translated versions of EQ-5D in four Indian languages (Hindi, Gujarati, Tamil, and Odia), which are used in the regions/states wherein the data collection was undertaken.

### Measurements

In this study, we have classified various socio-demographic variables based on their inherent characteristics. Age of respondents, number of dependent members in household, number of earning members in household, and monthly household income were considered as continuous variables. Continuous variables exhibit a continuous and unbroken spectrum of values, allowing for precise measurements. On the other hand, nominal variables, including gender, educational status, marital status, religion, area of residence, employment status, history of substance abuse, presence of ailments, province/state, and type of family, possess distinct categories or groups that do not have any inherent order or numeric value. These variables represent discrete attributes without a natural or meaningful sequence, hence used as nominal variables. Lastly, we treated the severity of the administered health state as an ordinal variable. Ordinal variables, unlike nominal ones, exhibit a meaningful order or hierarchy among their categories. In our study, the severity levels were ranked as very mild, mild, moderate, severe, and extreme. This classification of variables provided a comprehensive framework for analyzing and interpreting the diverse data collected in this study.

### Data analysis

As a single block of health-states (comprising health-states from the whole spectrum of severity) was valued by multiple individuals, each belonging to different socio-demographic characteristics, it offered an opportunity to assess the differential valuation of the similar health-states. First, the descriptive statistics were generated to present the characteristics of the final sample. The bivariate analysis was then performed using the value assigned to health-states (utility value) as the dependent variable, and socio-demographic factors as independent variables. This analysis was performed to assess the difference in the valuation of health-states across different socio-demographic groups. ANOVA was used to assess the presence of statistically significant differences between the mean utility scores among the respondents of different age-groups, states (provinces), occupation, religion, wealth quintile, substance abuse, presence of acute or chronic ailments, number of dependent members in the household, and severity of the administered health-states. On the basis of their severity, the health-states were classified into five groups, *viz.*, very mild, mild, moderate, severe, and extreme. This classification was based on the severity index/level sum score by adding up the levels of each dimension, where the best is (1 + 1 + 1 + 1 + 1) = 5, and the worst is (5 + 5 + 5 + 5 + 5) = 25. The independent samples *t*-test was used to see the difference in mean utility value among the respondents of different sex, area of residence, marital status, educational attainment, and family type (nuclear or joint).

The Tobit regression model was then used to ascertain the impact of different sociodemographic factors on the valuation of health-states. Tobit model was used as it is a class of regression model in which the observed range of the dependent variable is censored in some way (41–43). The utility value obtained as a result of health-state valuation was considered as the dependent variable, which was censored at −1. The socio-demographic factors were considered as independent variables. Those socio-demographic variables for which statistically significant differences were observed in the bivariate analysis among different groups were considered in the Tobit regression model. The regression equation thus formed is expressed as:


(1) $$Y^{*}=\beta_{0}+\beta_{1}X_{1}+\beta_{2}X_{2}+\ldots \beta_{k}X_{k}+e$$

where $X_{i}$ is the value of the *i*^th^ predictor, *e* is the error and $Y^{*}=\{\begin{array}{c} Y\;\mathrm{if}\;Y>-1\\ -1\;\mathrm{if}\;Y\leq -1 \end{array}$ .

### Ethical considerations

The study was performed in accordance with the ethical standards as laid down in the 1964 Declaration of Helsinki. The participants were presented the study’s participant information sheet, and written informed consent was obtained prior to the conduct of interview. The ethical approval to conduct the study was obtained from the Institutional Ethics Committee of the institute of affiliation of the corresponding author.

## Results

### Sample characteristics

A total of 2,311 interviews were considered in the final analysis after removing the incomplete/practice/pilot interviews and interviews flagged due to respondents’ lack of understanding. The interviews which were not included in the final analysis were predominantly pilot interviews (*N* = 788). Such a large pilot was conducted to ensure protocol compliance and minimize the interviewers’ effect, considering the limited literacy rate of the Indian population, and to standardize the data collection process across all the study sites as well as interviewers. The remaining interviews which were not included in the analysis were either incomplete (*N* = 98) or were requested for non-inclusion by the respondents because of their lack of understanding (*N* = 301), or were flagged by the interviewers due to the respondents’ lack of involvement (*N* = 50). The mean age of the respondents was 42 years (standard deviation: 16 years), the age ranged between 18 and 82 years old. Females comprised 51.1% of the sample. Majority of the respondents were married (70.6%) and resided in rural areas (68.7%). The detailed socio-demographic information of the respondents is presented in Table 1.

**Table 1:** Socio-demographic characteristics of the respondents.

Characteristics		Number	Percentage
Age group (Years)	<20	97	4.2
	20–29	625	27.0
	30–39	518	22.4
	40–49	467	20.2
	50–59	326	14.1
	60–69	188	8.1
	70+	90	3.9
Gender	Male	1,129	48.9
	Female	1,178	51.1
Educational status	Illiterate	250	10.8
	Primary	296	12.8
	Middle	395	17.1
	Matric	438	19.0
	Senior secondary	405	17.5
	Graduate and above	527	22.8
Marital status	Married	1,631	70.6
	Never married	498	21.5
	Widow/Divorced	182	7.9
Dependent members in household	One	218	9.4
	2–3	1,061	45.9
	4–5	762	33.0
	More than 5	270	11.7
Area of residence	Urban	724	31.3
	Rural	1,587	68.7
Employment status	Non-employed	1,176	50.9
	Self-employed	734	31.8
	Employed in public sector	178	7.7
	Employed in private sector	223	9.6
Substance abuse	Alcohol	115	5.0
	Tobacco (smoking/smokeless)	623	27.0
	Both alcohol and tobacco	33	1.4
	None	1,540	66.6
Presence of ailments	No ailment	1,362	58.9
	Chronic ailment	371	16.1
	Acute ailment	320	13.8
	Both acute and chronic ailments	258	11.2
Religion	Hindu	2,046	88.5
	Muslim	119	5.1
	Christian	115	5.0
	Other	31	1.3
Earning members in household	Single earning member	1,172	50.7
	Multiple earning members	1,139	49.3
Province/State	Haryana	432	18.7
	Gujarat	394	17.0
	Odisha	509	22.0
	Tamil Nadu	460	19.9
	Uttar Pradesh	516	22.3
Total		2,311	100

### Impact of sociodemographic factors on valuation of health

Bivariate analysis demonstrated that differences exist in the valuation of health across different groups of age, religion, family type, state of residence, substance abuse, presence of ailments at the time of valuation, and number of dependent members in the household (Table 2). We also found that the valuation of health was not different among respondents of different sex, wealth quintiles (based on monthly household income), area of residence (rural/ urban), marital status, level of educational attainment, occupation, and with different number of earning members in the household.

**Table 2:** Valuation of health across different socio-demographic groups.

Socio-demographic variables		Mean utility score	SD	F/t-ratio	*p* value
Age group (Years)	<20	0.159	0.617	0.304	**<0.01**
	20–29	0.171	0.62		
	30–39	0.173	0.625		
	40–49	0.142	0.625		
	50–59	0.142	0.62		
	60–69	0.124	0.634		
	>70	0.137	0.621		
Area of residence	Urban	0.144	0.635	0.928	0.354
	Rural	0.153	0.618		
Marital status	Married	0.151	0.623	0.253	0.801
	Unmarried	0.148	0.624		
Sex	Male	0.149	0.623	0.039	0.969
	Female	0.15	0.624		
Earning members in household	Single	0.155	0.621	1.248	0.212
	Multiple	0.145	0.625		
Occupation	Unemployed	0.155	0.616	2.34	0.072
	Self-employed	0.14	0.629		
	Employed in public sector	0.178	0.63		
	Employed in private sector	0.138	0.636		
Education	Illiterate	0.158	0.603	0.672	0.501
	Literate	0.149	0.626		
Religion	Hindu	0.155	0.621	14.27	**<0.01**
	Muslim	0.135	0.619		
	Christian	0.046	0.677		
	Other	0.253	0.525		
Wealth quintile (Monthly household income)	WQ 1	0.152	0.617	1.13	0.34
	WQ 2	0.154	0.622		
	WQ 3	0.135	0.631		
	WQ 4	0.148	0.621		
	WQ 5	0.161	0.625		
Substance abuse	None	0.159	0.619	6.01	**<0.01**
	Only Alcohol	0.125	0.659		
	Tobacco (smoking/smokeless)	0.138	0.622		
	Alcohol and Tobacco	0.039	0.681		
Presence of ailments	No ailment	0.167	0.619	9.94	**<0.01**
	Chronic ailment	0.128	0.634		
	Acute ailment	0.137	0.624		
	Both acute and chronic ailments	0.106	0.624		
Number of dependent members in Household	0–1	0.107	0.668	9.27	**<0.01**
	3–4	0.148	0.627		
	4–5	0.147	0.611		
	>5	0.2	0.6		
Family type	Nuclear	0.139	0.639	2.154	**0.031**
	Joint	0.157	0.613		
State/Province	Haryana	0.273	0.526	71.47	**<0.01**
	Gujarat	0.14	0.665		
	Odisha	0.145	0.627		
	Tamil Nadu	0.061	0.69		
	Uttar Pradesh	0.152	0.588		
Severity of health state	Very Mild	0.912	0.11	10,090	**<0.01**
	Mild	0.469	0.362		
	Moderate	0.168	0.461		
	Severe	−0.257	0.452		
	Extreme	−0.777	0.249		

Bold entries indicate significant difference at *a* = 0.05; SD, Standard deviation; WQ, Wealth quintile.

The results of Tobit regression model showed that even after controlling for the severity of the administered health states, factors having a significant impact on the valuation of health are age, religion, state/ province of residence, substance abuse, family type, number of dependent members in the household, and presence of chronic or both acute and chronic ailments (Table 3). The results demonstrated that younger individuals place a slightly better/higher value to the same health-state as compared to their older counterparts, although the magnitude of difference is less (*B* = −0.001). This is to mention here that assigning a better/higher value to a health-state implies that the individual perceives this health state to be less troublesome, as compared to another person who is assigning a lower value to it. It was further observed that people living in joint family rates the same health state as relatively worse (*B* = −0.016), as compared to people living in nuclear families. It was also observed that as compared to a healthy individual, a person with chronic (*B* = −0.051), or both acute and chronic ailments (*B* = −0.059), assigns a lower value the same health-state, thus rating it worse. Likewise, as compared to an individual with no history of substance abuse, a person consuming tobacco (*B* = −0.02), alcohol (*B* = −0.052), or both (*B* = −0.086) assigns a lower value the same health-state. It has also been observed that people living in Gujarat and Tamil Nadu rates the same health state as worse, as compared to people living in Odisha and Uttar Pradesh (44). The detailed results have been presented in Table 3.

**Table 3:** Impact of socio-demographic factors on valuation of health.

Variables	Coefficient	SE	*t* value	*p* value	95% confidence interval	
Constant	1.086	0.013	84.800	**<0.001**	1.061	1.111
Age	−0.001	0.000	−5.180	**<0.001**	−0.001	−0.001
Number of dependent members in household	0.004	0.002	2.360	**0.018**	0.001	0.007
Substance abuse (Ref: None)						
Alcohol	−0.044	0.012	−3.730	**<0.001**	−0.067	−0.021
Tobacco	−0.017	0.006	−2.900	**0.004**	−0.029	−0.006
Both alcohol and tobacco	−0.079	0.021	−3.730	**<0.001**	−0.120	−0.037
Presence of ailments (Ref: No ailment)						
Chronic ailment	−0.048	0.008	−6.430	**<0.001**	−0.063	−0.034
Acute ailment	−0.009	0.008	−1.160	0.247	−0.024	0.006
Both acute and chronic ailments	−0.056	0.009	−6.090	**<0.001**	−0.074	−0.038
Religion (Ref: Hindu)						
Muslim	0.004	0.011	0.310	0.754	−0.019	0.026
Christian	−0.063	0.012	−5.420	**<0.001**	−0.086	−0.041
Other	0.059	0.022	2.740	**0.006**	0.017	0.102
Family type (Ref: Nuclear family)						
Joint family	−0.015	0.006	−2.400	**0.017**	−0.027	−0.003
Province/state (Ref: Haryana)						
Gujarat	−0.157	0.009	−17.950	**<0.001**	−0.174	−0.140
Odisha	−0.108	0.008	−13.100	**<0.001**	−0.125	−0.092
Tamil Nadu	−0.201	0.009	−22.200	**<0.001**	−0.219	−0.183
Uttar Pradesh	−0.109	0.009	−12.360	**<0.001**	−0.126	−0.092
Severity of health states (Ref: Very mild)						
Mild	−0.443	0.008	−54.040	**<0.001**	−0.459	−0.427
Moderate	−0.750	0.008	−99.960	**<0.001**	−0.765	−0.735
Severe	−1.178	0.008	−141.340	**<0.001**	−1.195	−1.162
Extreme	−1.688	0.009	−178.580	**<0.001**	−1.707	−1.670

Bold entries indicate significant difference at *a* = 0.05. SE, Standard error.

Older individuals have a slight negative valuation of health (Table 3). It implies that if a person with an older age encounters a health condition, it is more likely to be reported, and with increased severity, as compared to a person in young age. One possible reason for this can be that in younger age, health is not as much a priority as ensuring job security, earning livelihood, and fulfilling social belonging needs (45). With increasing age, as other age-specific needs and societal responsibilities reach their culmination, the health starts to be recognized as a priority. Moreover, ill health leads to a disproportionately higher disability or poor quality of life among the older adults, and hence have a higher aversion to ill health and consequently, poorer reported quality of life for same health state. Another possible explanation of the age-specific association is that with higher age, an individual is more likely to have been influenced by illnesses, and hence rates the health low.

Furthermore, it has been observed in our study that a person suffering from ailments or doing substance abuse assigns a lower value to the same health-state as compared to a healthy individual. This explains that due to the experience of morbidity, and individual becomes more conscious and aware of its consequences, hence a small decrement in health is seen and reported with a greater magnitude. It has also been observed in our study that when a health condition is encountered by a person living in joint family, it perceives it worse as compared to a person living in nuclear family. This could be because joint families usually have a better support system to take care of the personal, health, and financial needs during the morbidity, hence placing the health higher on their priority list (46). Likewise, the impact of an increase in the number of dependent members in the household on the valuation of health can be explained in the same way.

## Discussion

The empirical estimation of the self-reported morbidity rate is a manifestation of two things. The first component is a consequence of the biomedical phenomenon which depends on the actual health status of the members of a socioeconomic group. Therefore, biologically, there can be a differential prevalence of illnesses in rich and poor populations. The second component rests upon the propensity of reporting the existing illness, which has a philosophical basis and has been explained with the help of positional objectivity (12). Therefore, the empirically measured rates of self-reported morbidity in the rich and poor populations could have been contributed by the way rich/poor populations perceive ill health (differential valuation of health). As certain surveys report that the self-reported morbidity rate is higher among the wealthier quintiles of the population, and as it goes contrary to the objective measurements of health, it has been hypothesized that this phenomenon could be a result of positional objectivity (47). In this paper, we have investigated the role of positional objectivity in confounding the self-reported morbidity rate. Our study has investigated that when people from different sociodemographic backgrounds encounter a same health condition, do they place a different value to it.

The findings of our research implies that as there is no role of positional objectivity in health, empirically estimated self-reported morbidity rate is a consequence of the actual health status of a person. This being the case, the differential reporting of ill health among the rich and the poor is predominantly influenced by the prevalence of communicable and non-communicable diseases in these population sub-groups. As the incidence of communicable diseases has a strong correlation with social determinants of health, these diseases are more likely to be concentrated in poor population (48). On the other hand, the concentration of non-communicable diseases across the wealth quintiles is dependent on the phase of epidemiological transition in the respective populations (15). If the population is in an early phase of epidemiological transition, then the non-communicable diseases tend to be more prevalent in the high-income groups. Whereas, with the advancement of the epidemiological transition, the lower income groups of a population also start to face increased prevalence of non-communicable diseases. If positional objectivity is not contributing to the differential rate of self-reported morbidity among the rich and poor population as our findings suggest, then this differential is an interplay of distribution of communicable and non-communicable diseases across these population sub-groups. In the early phases of epidemiological transition, the self-reported morbidity rates are result of the competing effect of higher prevalence of communicable diseases in poor population and higher prevalence of non-communicable diseases in the rich population. When we move to areas with established epidemiological transition, the poor population start to face the dual burden of disease, as not only the communicable diseases are more prevalent in the poorer population, but the prevalence of non-communicable diseases also starts to rise in these population subgroups. As a consequence, there would be higher income-based inequalities in the health status of those population groups where the epidemiological transition has set in.

We have also investigated what other factors impact the differential valuation of health. Using the empirical data from India, this study is the first attempt to demonstrate the differential valuation of health by the different strata of the society. Overall, our findings suggest that the value which a person assigns to the health is influenced by an array of sociodemographic factors like its age, religion, state/province of residence, substance abuse, family type, number of dependent members in their household, and presence of chronic or both acute and chronic ailments.

Our findings demonstrate that increasing age has a negative influence on the valuation of health. It implies that if a person with an older age encounters a health condition, he/she is more likely to report it, and with increased severity, as compared to a person in younger age. This explains that with increasing age, a person becomes more conscious and aware about its health, as compared to its younger counterparts. One potential reason for this can be that in the younger age, health is not as much a priority as ensuring job security, earning livelihood, and fulfilling social belonging needs, as reported by Abdi et al. (45). This implies that with increasing age, as other age-specific needs and societal responsibilities reach their culmination, the health starts to be recognized as a priority.

Likewise, it has been observed in our analysis that a person suffering from ailments or doing substance abuse assigns a lower value to the same health-state as compared to a healthy individual. This explains that due to the experience of morbidity, and individual becomes more conscious and aware of its consequences, hence a small decrement in health is seen and reported with a greater magnitude (49). It has also been observed that when a health condition is encountered by a person living in joint family, it perceives it worse as compared to a person living in nuclear family. This is because joint families usually have a better support system to take care of the personal, health, and financial needs during the morbidity, hence placing the health higher on their priority list, which has also been demonstrated by Gupta et al. (46). The impact of an increase in the number of dependent members in the household on the valuation of health can also be explained in the same way, which corresponds to the findings by O’Gara et al. (50).

It is noteworthy to highlight that our study yielded an intriguing finding, as we did not observe any statistically significant disparities in the valuation of health among individuals across various wealth quintiles, as depicted in Table 2. This outcome stands in contrast with the hypotheses of positional objectivity proposed by Amartya Sen that posited individuals from lower-income strata tend to underreport or underestimate the presence of illness or health deficits (2, 12, 13). Hence, the findings of our study challenges conventional assumptions proposed in the literature regarding the relationship between socioeconomic status and health perception. However, it is important to note that this hypothesis of positional objectivity has faced contrary findings in subsequent analyses by Subramanian et al. (9) that examined the relationship between socio-economic status and the prevalence of self-reported morbidities in India. Their investigations have revealed that individuals from lower socio-economic strata are more inclined to report specific health issues, illnesses, and an overall perception of poor health. Likewise, investigations by Dixit et al. and Gupta et al. on health outcomes on distinct patient groups in India have also failed to establish any discernible impact of income on self-reported health outcomes (46, 51). In essence, the research landscape has yielded a spectrum of results, encompassing positive, null, and negative associations between income inequality and self-reported health (52, 53). Within this context, our analysis contributes to the body of knowledge by demonstrating a lack of association between an individual’s wealth status and their inclination to assign varying values to their health.

A possible limitation while examining positional objectivity in our study might be that the wealth status of an individual was ascertained on the basis of self-reported household income, which could be under-reported. As income is a sensitive and private topic, the income related questions asked in the context of survey research are very susceptible to misreporting (54). This could become more pronounced in the Indian settings where the proportion of informal sector employment predominates in the labor workforce (55). As a result, information on income can be under-reported. However, as long as the extent of under-reporting is random among the population sub-groups, it will not confound our analysis. Even if the under-reporting is systematic, and higher among the rich, it may affect the gradient of health reporting among the intermediate socio-economic status groups, but the comparison of extreme groups should demonstrate significant difference. We did not find any significant difference between the poorest and the richest. Moreover, the direction of valuation was also opposite to what could be expected as a result of positional objectivity. Finally, other proxy measures of socio-economic status such as education or occupation also had a null association.

## Conclusion

In conclusion, our investigation has probed the impact of positional objectivity in confounding the self-reported morbidity rates. We have examined whether individuals from diverse sociodemographic backgrounds assign varying values to the same health conditions. Our findings underscore that positional objectivity does not play a role in health valuation, ultimately indicating that empirically estimated self-reported morbidity rates directly reflect an individual’s actual health status. Consequently, disparities in reporting ill health between affluent and less affluent individuals primarily arise from variations in the prevalence of communicable and non-communicable diseases within these subpopulations. Our study reveals that an individual’s sociodemographic context and subsequent social experiences shape their health valuations, with household income showing no discernible impact on such assessments. Hence, disparities in empirically estimated self-reported morbidity rates among diverse socio-economic groups inherently stem from the genuine health conditions within these population segments. The results of this study can be used in understanding inequalities in self-reported morbidities and consequently fine-tune the policy initiatives aimed at specific demographic groups by understand their differential health needs.

## Data availability statement

The original contributions presented in the study are included in the article/supplementary material, further inquiries can be directed to the corresponding author.

## Ethics statement

The studies involving humans were approved by the Institutional Ethics Committee of Postgraduate Institute of Medical Education and Research, Chandigarh, India, vide reference no. PGI/IEC/2018/001629. The studies were conducted in accordance with the local legislation and institutional requirements. The participants provided their written informed consent to participate in this study.

## Author contributions

GJ and SP: conception and design of the work. GJ and SP: data collection. GJ, SP, AG, BG, MK, and SG: data analysis and interpretation. GJ and SP: drafting the article. GJ, SP, AG, BG, MK, and SG: critical revision of the article. All authors contributed to the article and approved the submitted version.
